# Presynaptic Localization and Possible Function of Calcium-Activated Chloride Channel Anoctamin 1 in the Mammalian Retina

**DOI:** 10.1371/journal.pone.0067989

**Published:** 2013-06-26

**Authors:** Ji Hyun Jeon, Sun Sook Paik, Myung-Hoon Chun, Uhtaek Oh, In-Beom Kim

**Affiliations:** 1 Department of Anatomy, College of Medicine, The Catholic University of Korea, Seoul, Korea; 2 Catholic Neuroscience Institute, College of Medicine, The Catholic University of Korea, Seoul, Korea; 3 Channel Research Center, College of Pharmacy, Seoul National University, Seoul, Korea; 4 Catholic Institute for Applied Anatomy, College of Medicine, The Catholic University of Korea, Seoul, Korea; Virginia Tech Carilion Research Institute, United States of America

## Abstract

Calcium (Ca^2+^)-activated chloride (Cl^−^) channels (CaCCs) play a role in the modulation of action potentials and synaptic responses in the somatodendritic regions of central neurons. In the vertebrate retina, large Ca^2+^-activated Cl^−^ currents (I_Cl(Ca)_) regulate synaptic transmission at photoreceptor terminals; however, the molecular identity of CaCCs that mediate I_Cl(Ca)_ remains unclear. The transmembrane protein, TMEM16A, also called anoctamin 1 (ANO1), has been recently validated as a CaCC and is widely expressed in various secretory epithelia and nervous tissues. Despite the fact that *tmem16a* was first cloned in the retina, there is little information on its cellular localization and function in the mammalian retina. In this study, we found that ANO1 was abundantly expressed as puncta in 2 synaptic layers. More specifically, ANO1 immunoreactivity was observed in the presynaptic terminals of various retinal neurons, including photoreceptors. I_Cl(Ca)_ was first detected in dissociated rod bipolar cells expressing ANO1. I_Cl(Ca)_ was abolished by treatment with the Ca^2+^ channel blocker Co^2+^, the L-type Ca^2+^ channel blocker nifedipine, and the Cl^−^ channel blockers 5-nitro-2-(3-phenylpropylamino) benzoic acid (NPPB) and niflumic acid (NFA). More specifically, a recently discovered ANO1-selective inhibitor, T16A_inh_-A01, and a neutralizing antibody against ANO1 inhibited I_Cl(Ca)_ in rod bipolar cells. Under a current-clamping mode, the suppression of I_Cl(Ca)_ by using NPPB and T16A_inh_-A01 caused a prolonged Ca^2+^ spike-like depolarization evoked by current injection in dissociated rod bipolar cells. These results suggest that ANO1 confers I_Cl(Ca)_ in retinal neurons and acts as an intrinsic regulator of the presynaptic membrane potential during synaptic transmission.

## Introduction

Calcium (Ca^2+^)-activated chloride (Cl^−^) channels (CaCCs) are anion-selective channels that are activated by increased cytosolic Ca^2+^. CaCCs have been implicated in many important physiological processes, such as the transepithelial transport of electrolytes and water, control of vascular tone, and cardiac muscle and neuronal excitability [1]–[4]. In the nervous system, Ca^2+^-activated Cl^−^ current (I_Cl(Ca)_) is primarily observed in primary sensory neurons, such as olfactory receptor neurons (ORNs), taste receptor cells, somatosensory neurons of dorsal root ganglia (DRG), and photoreceptors of the retina, and is involved in corresponding sensory transduction. I_Cl(Ca)_ is also found in presynaptic terminals in the brain, where it is thought to modulate synaptic activity [1].

Anoctamin 1 (ANO1, also called TMEM16A) [5]–[7] is a CaCC because its biophysical and pharmacological characteristics correspond to those of endogenous CaCCs [8], [9]. The identification of ANO1 as a CaCC has unveiled its significance in many physiological activities, including (1) Cl^−^ transport in airways [5], [10], [11], salivary glands [7], [12], and gastrointestinal epithelial cells [13], [14], (2) rhythmic contraction in gastrointestinal tracts [13]–[15], and (3) heat sensation in DRG neurons [16].

The retina is a well-characterized model system that is used for the study of synaptic mechanisms, as it contains various neurotransmitters found in the central nervous system and its receptors; various types of synapses, such as conventional chemical, electrical, and distinct ribbon synapses; and several well-established synaptic circuits for visual processing. I_Cl(Ca)_ has been characterized in the photoreceptors of the vertebrate retina [17], [18] and is thought to regulate synaptic transmission at photoreceptor terminals by stabilizing membrane potentials and Ca^2+^ channel modulation [19]–[24]. Recently, ANO1 [25] and ANO2 [26], which is another anoctamin with CaCC characteristics [27], were identified in photoreceptor terminals in salamander and mouse retinas, respectively. They are thought to be strong candidates for the molecular identity of I_Cl(Ca)_ in photoreceptors. However, their specific functions in photoreceptors are not known in the mammalian retina. In addition, I_Cl(Ca)_ has been identified in goldfish bipolar cells [28], which are another type of retinal neuron. These findings suggest the presence of I_Cl(Ca)_ in other retinal neurons, even though its molecular identity and function remain unknown. Thus, we sought to examine the expression, localization, and function of ANO1 in the mouse retina.

## Results

### Expression and localization of ANO1 in the retina

**Figure 1:**
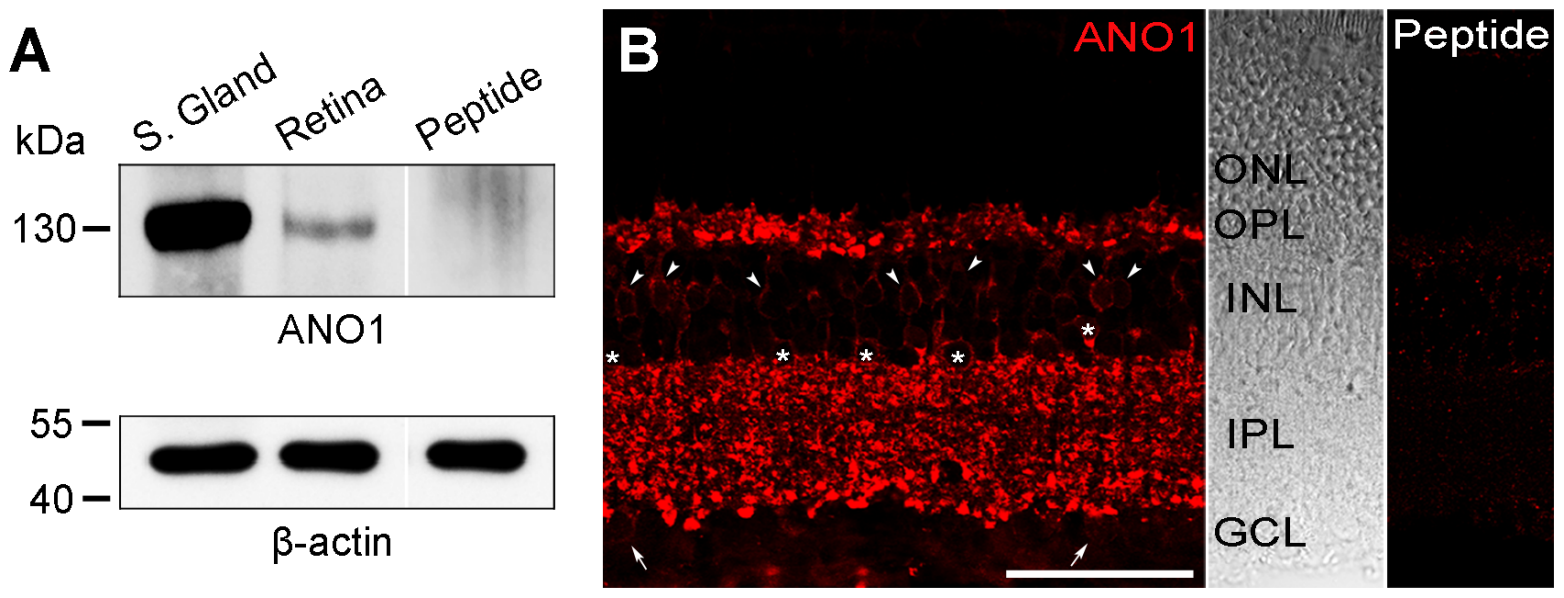
Expression of ANO1 in the mouse retina. **A**. Western blot analysis of ANO1 in mouse retina homogenates. Salivary gland extracts were used as a positive control. Preincubation of the anti-ANO1 antibody with a 10-fold excess (w/w) of the antigenic peptide led to an absence of bands. **B**. Confocal micrograph taken from a vertical vibratome section (50 µm in thickness) of the mouse retina processed for ANO1 immunoreactivity. Strong ANO1-immunoreactive puncta are shown in the OPL and IPL. Many bipolar (arrowheads) and amacrine cells (asterisks) in the INL and some (arrows) cells in the GCL exhibit weak ANO1 immunoreactivity. The DIC image presented in the middle shows the retinal layers. In the control experiment shown on the right, preincubation of the anti-ANO1 antibody with a 10-fold excess (w/w) of the antigenic peptide led to an absence of labeling. OPL, outer plexiform layer; IPL, inner plexiform layer; INL, inner nuclear layer; GCL, ganglion cell layer; ONL, outer nuclear layer; DIC, differential interference contrast. Scale bar, 50 µm.

To investigate the expression and distribution pattern of the ANO1 protein in the mouse retina, western blotting and immunohistochemistry were performed. As shown in Fig. 1A, an ANO1 immunoreactive band (∼130 kDa) was recognized in both the retina and salivary gland (the latter was used as a control tissue in which ANO1 is expressed abundantly) [7], [12]. Strong ANO1 immunoreactivity was observed as puncta in 2 synaptic layers, the outer plexiform layer (OPL) and the inner plexiform layer (IPL), whereas weak immunoreactivity was detected in some somata in the inner nuclear layer (INL) and ganglion cell layer (GCL). There was no immunoreactivity in the outer nuclear layer (ONL) (Fig. 1B).

**Figure 2:**
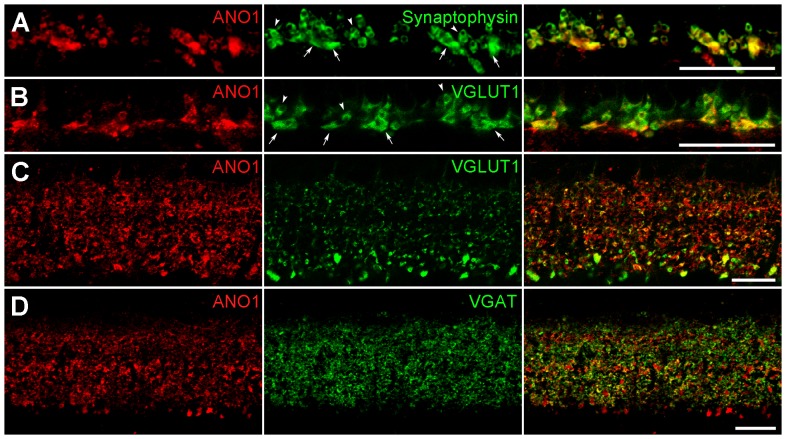
Cellular and subcellular localization of ANO1 in the retina. (**A, B**) Outer plexiform layer (OPL). **A**. Large and small ANO1-labeled puncta (red) in the OPL demonstrate synaptophysin immunoreactivity (green). 2 types of synaptophysin-labeled puncta (smaller higher-positioned rod spherules (arrowheads) and larger lower-positioned cone pedicles (arrows)) are clearly seen. **B**. Similar to **A**, the anti-VGLUT1 antibody (green) labels rod and cone terminals. In the merged image, smaller puncta with a round shape, putative rod spherules (arrowheads), and 4 larger puncta with a linear shape (arrows; putative cone pedicles) show ANO1 (red) and VGLUT1 immunoreactivities. (**C, D**) Inner plexiform layer (IPL). Numerous ANO1-labeled puncta (red) of various sizes are observed in the IPL. In **C**, the anti-VGLUT1 antibody (green) labels numerous bipolar axon terminals in the IPL. Some ANO1-immunoreactive puncta show VGLUT1 immunoreactivity. In **D**, VGAT immunoreactivity (green) is seen as tiny puncta throughout the IPL. ANO1 is partially colocalized with VGAT. Scale bars, 20 µm.

Next, we determined the cellular and subcellular localization of ANO1 in the retina via double-labeling experiments using various neuronal and synaptic markers. ANO1 puncta in the OPL, where photoreceptor terminals synapse onto bipolar and horizontal cell dendrites, showed immunoreactivities for synaptophysin and vesicular glutamate transporter 1 (VGLUT1), which are markers of photoreceptor terminals (Figs. 2A and B), but not for Goα, which is an ON bipolar cell marker (Fig. S1A), and calbindin, a horizontal cell marker (Fig. S1B). These results indicate that ANO1 is expressed in photoreceptor terminals, but not in bipolar and horizontal cell dendrites. In the IPL, where bipolar axon terminals synapse onto ganglion cell dendrites and amacrine processes, which in turn give synaptic inputs to ganglion dendrites and bipolar terminals, ANO1 was detected in bipolar cell terminals that were immunoreactive for VGLUT1, which is a bipolar terminal marker (Fig. 2C), and in amacrine cell processes that were immunoreactive for vesicular GABA transporter (VGAT), which is an amacrine terminal marker (Fig. 2D), but not in ganglion cell dendrites exhibiting immunoreactivity for SMI32, which is a ganglion cell marker (Fig. S1C).

**Figure 3:**
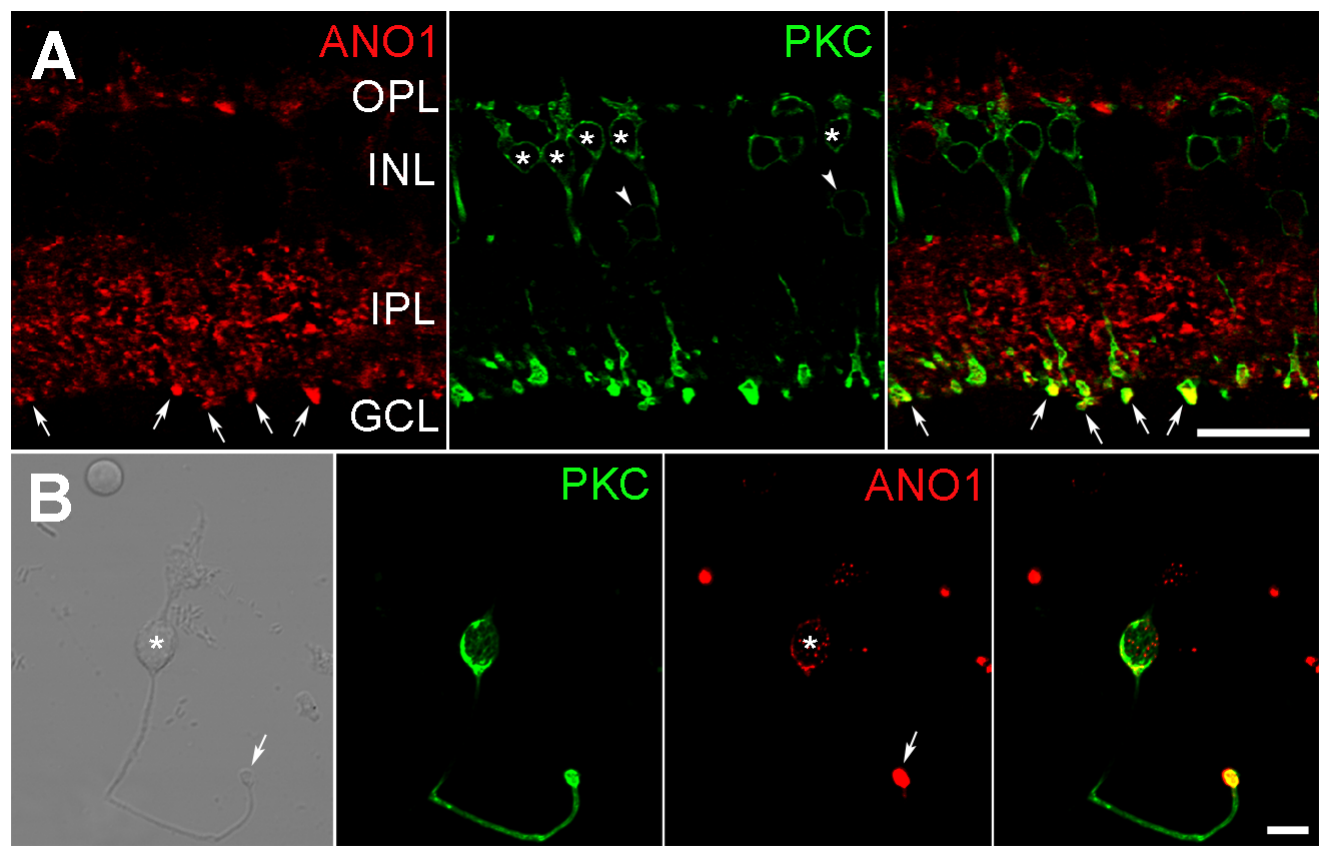
ANO1 expression in the rod bipolar cell. Confocal micrographs taken from a vertical vibratome section of the mouse retina and a dissociated bipolar cell processed for ANO1 (red) and PKC (green) immunostaining. **A**. Many ANO1-labeled puncta of various sizes are observed throughout the IPL. Note the large puncta (arrows) located in the innermost part of the IPL. PKC-labeled rod bipolar (asterisks) and amacrine (arrowheads) cell somata are found in the INL, and PKC-labeled axon terminals are clearly seen in the IPL, close to the GCL. Large ANO1 puncta (arrows) are located in the PKC-labeled rod bipolar axon terminals. **B**. DIC image showing a dissociated retinal bipolar cell with a large axon terminal (arrow). The asterisk indicates its soma. The dissociated bipolar cell shows PKC immunoreactivity, and ANO1 immunoreactivity is seen strongly in the axon terminal (arrow) and weakly in the soma (asterisk). The merged image shows that ANO1 is expressed in a PKC-labeled rod bipolar cell, and that its expression is especially strong in the axon terminal. Scale bars, 20 µm (**A**) and 5 µm (**B**).

In vertical retinal sections, large and strongly labeled ANO1 puncta exhibiting VGLUT1 immunoreactivity were observed in the IPL, close to the GCL (Fig. 2C). This is where stratification of the axon terminals of rod bipolar cells, which are a subpopulation of second-order neurons involved in processing scotopic vision [29], occurs. Thus, we confirmed that ANO1 is expressed in rod bipolar axon terminals in retinal slices (Fig. 3A) and dissociated cells (Fig. 3B), as assessed via double-labeling of ANO1 and PKC, which is a marker for rod bipolar cells. Taken together, our results suggest that ANO1 is expressed in various retinal neurons, including rod bipolar cells, and that ANO1 is preferentially localized to the presynaptic region.

**Figure 4:**
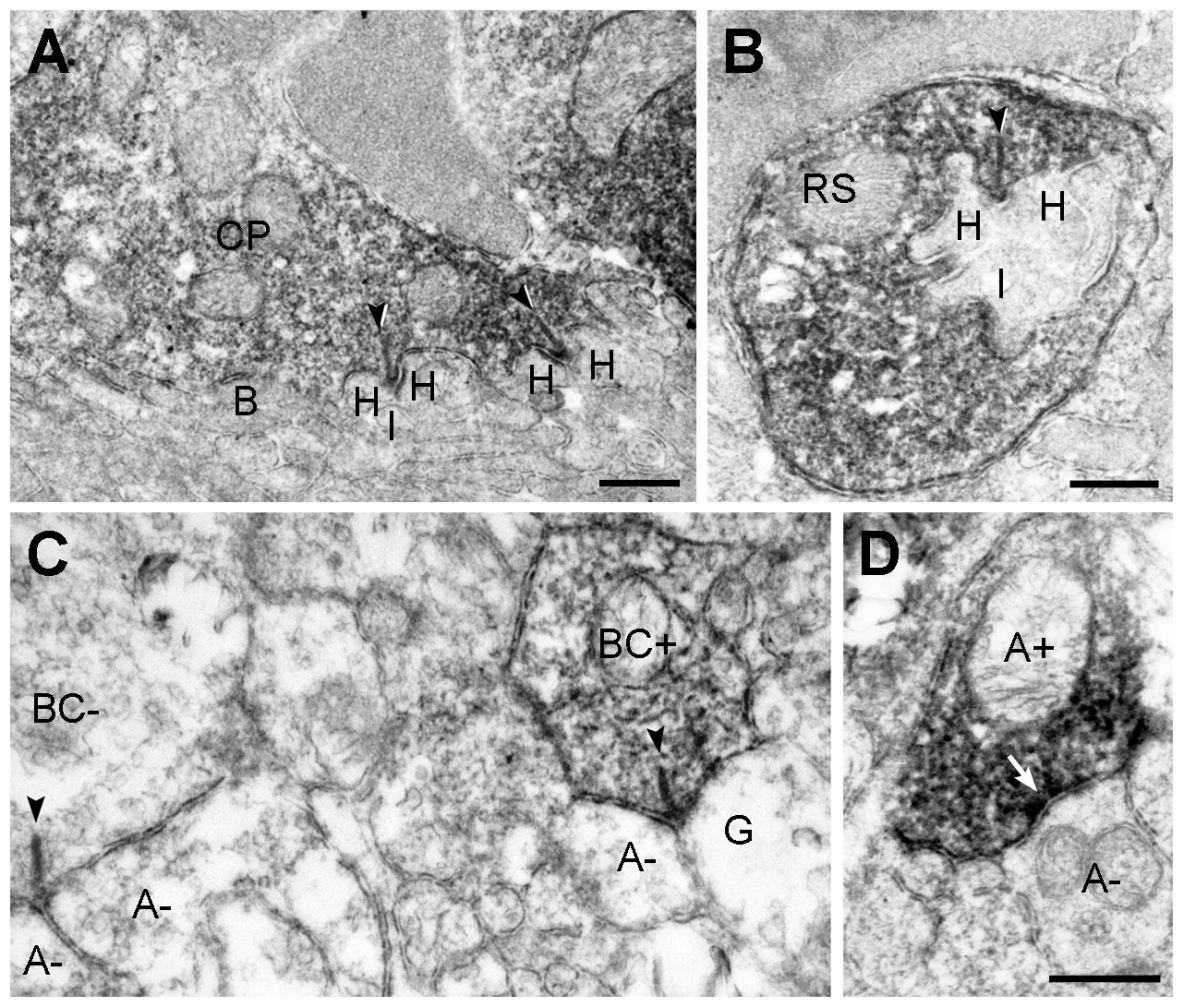
Presynaptic localization of ANO1 in the retina. Electron micrographs taken from vertical ultrathin sections (90 nm in thickness) of the mouse retina processed for ANO1 immunostaining. (**A, B**) Outer plexiform layer. In **A**, ANO1 immunoreactivity is localized to a cone pedicle (CP). At each ribbon synapse (arrowheads) within the CP, 2 horizontal dendrites (H) and an invaginating dendrite of ON-cone bipolar cell (I) are unlabeled. The basal dendrites of OFF-cone bipolar cells (B) at the CP base are also unlabeled. In **B**, a rod spherule (RS) shows ANO1 immunoreactivity. Similar to **A**, the postsynaptic triad comprising 2 horizontal dendrites (H) and an invaginating rod bipolar dendrite (I) do not exhibit ANO1 immunoreactivity. (**C, D**) Inner plexiform layer. In right side of **C**, a labeled cone bipolar terminal (BC+) establishes a ribbon synapse (arrowhead) onto a postsynaptic dyad composed of an unlabeled ganglion dendrite (G) and an unlabeled amarcrine process (A−). In addition, an unlabeled bipolar terminal (BC−) synapsing onto 2 unlabeled amacirne dendrites (A−) is seen in the left side. In **D,** a labeled amacrine process (A+) establishes a conventional chemical synapse onto an unlabeled amacrine process (A−). Scale bars, 0.5 µm.

We used pre-embedding immunoelectron microscopy to confirm the presynaptic localization of ANO1 in retinal neurons. In the OPL, cone pedicles and rod spherules were labeled for ANO1, whereas invaginating ON-bipolar and horizontal cell dendrites, which are postsynaptic elements located at the ribbon synapse of photoreceptor terminals, and OFF-bipolar cell dendrites, which make basal junctions with cone pedicles, were unlabeled (Figs. 4A and B). In the IPL, ANO1 labeling was observed in some bipolar terminals (Fig. 4C), including rod bipolar cells, which were identified by their synaptic ribbons, and amacrine processes, which were filled with synaptic vesicles and established conventional chemical output synapses (Fig. 4D). However, ganglion cell dendrites, which contain microtubules and microfilaments instead of synaptic vesicles, were not immunolabeled (Fig. 4C). These results were consistent with confocal microscopy findings, as shown in Figs. 2 and 3, and confirmed the preferential presynaptic localization of ANO1 in various retinal neurons.

### Identification and characterization of I_Cl(Ca)_ in rod bipolar cells expressing ANO1

Our anatomical findings strongly imply the presence of ANO1 in rod bipolar cells. However, the presence and functions of CaCCs and/or I_Cl(Ca)_ mediated by CaCCs have not been reported in rod bipolar cells in the mammalian retina (however, please refer to a previous report of I_Cl(Ca)_ in a bipolar cell type in the goldfish retina [28]). Thus, we aimed to test whether I_Cl(Ca)_ is present in mouse rod bipolar cells, and to characterize this current.

**Figure 5:**
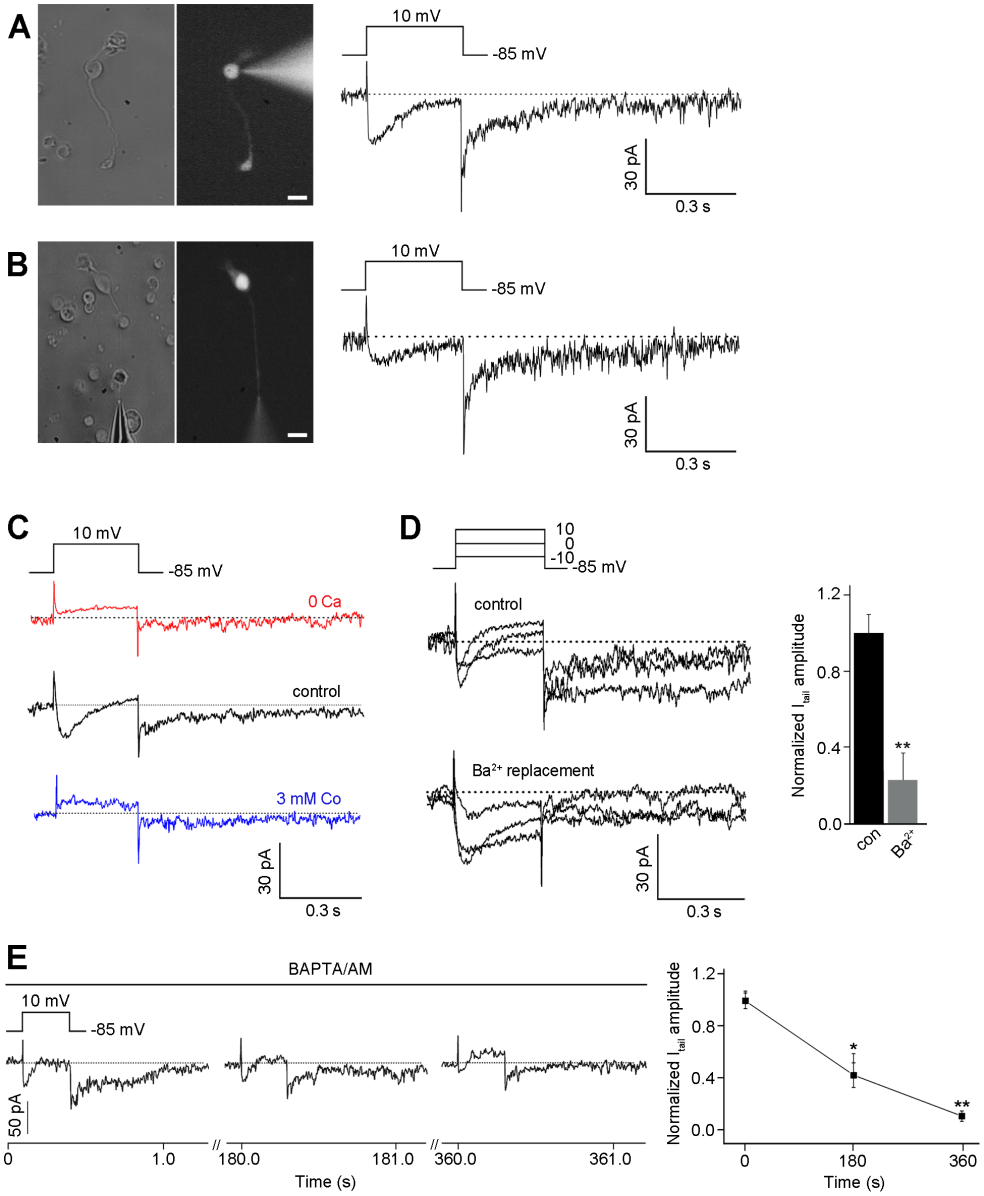
I_tail_ recorded in the rod bipolar cell is activated by Ca^2+^ influx. (**A, B**) The cells that retained their axon terminals were filled with Lucifer Yellow during recording and were morphologically identified under a fluorescence microscope after recording (left panel). The inwardly generating I_Ca_ was recorded from a voltage-clamped bipolar cell with a retained axon terminal at the voltage of +10 mV from a holding potential of −85 mV; subsequently, I_tail_ was activated when returned to the holding potential. **C**. A solution containing zero Ca^2+^, a control solution (5 mM Ca^2+^), and a solution of 3 mM Co^2+^ were applied serially (n = 13). Currents were recorded from a voltage-clamped bipolar cell with a retained axon terminal at the voltage of +10 mV from a holding potential of −85 mV. **D**. Ca^2+^ was replaced with Ba^2+^ in the extracellular solution (n = 10). **E**. BAPTA/AM (0.1 mM), which is a membrane-permeable Ca^2+^ chelator, was applied extracellularly to show the time course of changes in I_tail_. Scale bars, 5 µm.

Whole-cell currents were recorded from somata (Fig. 5A; n = 20) and axon terminals (Fig. 5B; n = 7) of freshly dissociated rod bipolar cells, including long axons and terminals. The kinetics of the 2 currents were almost identical, as seen in Fig. 5. Upon depolarizing the cells to the voltage of +10 mV from a holding potential of −85 mV, inward current was generated, followed by a slowly inactivating tail current (I_tail_) (Figs. 5A and B). This inward current was identified as L-type Ca^2+^ current (I_Ca_), which was described previously in rod bipolar cells [30], [31], because (1) potassium currents and h-current were suppressed by tetraethylammonium chloride (TEA) and CsCl; (2) it was detectable at depolarizing potentials between about −60 mV and +40 mV and had a peak amplitude of 20±4 pA at a voltage of about −30 mV; and (3) it was sensitive to nifedipine, which is L-type Ca^2+^ channel antagonist (Fig. S2). I_tail_ was also detectable; its activation started at about −30 mV and peaked at about +10 mV. The maximum amplitude was ∼30 pA and I_tail_ lasted for ∼1 s.

To test whether the activation of I_tail_ was dependent on I_Ca_, a solution containing no Ca^2+^ and 3 mM Co^2+^ (Ca^2+^ channel blocker) were applied in series as shown in Fig. 5C (n = 13). In this series of recordings, both I_Ca_ and I_tail_ were not found in the absence of Ca^2+^, were recovered in control solution, and were suppressed again in the presence of 3 mM Co^2+^. Next, we tested whether Ba^2+^ activated I_tail_ as a carrier that permeates the Ca^2+^ channels (Fig. 5D; n = 10). I_tail_ was abolished by the replacement of external Ca^2+^ with equimolar Ba^2+^, whereas I_Ca_ was enhanced because of Ba^2+^ conductance. Moreover, the intracellular [Ca^2+^]_i_ dependency of I_tail_ was tested by the application of Ca^2+^ chelators in the pipette solution. The introduction of both BAPTA (> 10 mM) (n = 8) and EGTA (5 mM) (n = 8) into bipolar cells via a recording pipette strongly suppressed I_tail_ (Fig. S3). Furthermore, we applied BAPTA/AM (0.1 mM), which is a membrane-permeable Ca^2+^ chelator, extracellularly (Fig. 5E; n = 8). During the treatment with BAPTA/AM, the peak amplitude (40.3±8.1%) of I_tail_ decreased, and this current disappeared almost completely after ∼6 min (7.3±4.2%); in contrast, I_Ca_ remained unchanged. These experiments demonstrate that I_tail_ of rod bipolar cells is activated by increased [Ca^2+^]_i_.

**Figure 6:**
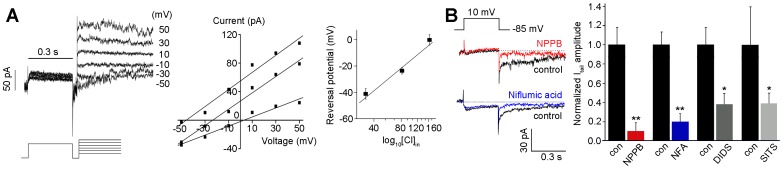
Cl^−^ ions are identified as the ionic component of I_tail_. **A**. The reversal potential of I_tail_ was measured at 3 different [Cl^−^]_i_: 144 mM, 1.9±1.7 mV (n = 16); 72 mM, −23.6±4.2 mV (n = 12); 29 mM, −46.2±1.1 mV (n = 17). The peak amplitude of I_tail_ was measured 10 ms after the command pulse, to avoid recording interference from the capacitative current. The average reversal potentials, which were plotted as a function of log_10_ [Cl^−^]_i_, fitted to the values estimated from the Nernst equation with a slope of ∼−58 mV. **B**. The NPPB (100 µM), NFA (100 µM), DIDS (1 mM) and SITS (2 mM) were applied. The results of statistical analyses are presented in the panel on the right as the normalized mean ± S.D.. Student’s *t* tests were used to compare the data from the 2 groups. Significance was set as *P*<0.05 (*) and *P*<0.01 (**).

To identify the ionic component of I_tail_, we measured the reversal potential of I_tail_ at 3 different [Cl^−^]_i_; 144 mM, 72 mM and 29 mM (Fig. 6A). As either standard or reduced [Cl^−^]_i_ supplemented with methanesulfonate, the reversal potential of I_tail_ followed the shift in the equilibrium potential of Cl^−^ (E_Cl_) (Fig. 6A, middle panel). The average reversal potentials, which were plotted as a function of log_10_ [Cl^−^]_i_, fitted to the values estimated from the Nernst equation with a slope of ∼ −58 mV (Fig. 6A, right panel). Thus, the major component of this I_tail_ was identified as a Cl^−^ current. However, this Cl^−^ conductance may have been due to the activation of glutamate transporters in the retina [32]. To rule out this possibility, we applied a glutamate transporter inhibitor, dl-threo-β-benzyloxyaspartate (dl-tBOA). Blocking the glutamate transporter did not induce a significant change in I_tail_ (n = 8; data not shown), suggesting that the portion of Cl^−^ conductance that is mediated by the this transporter is negligible in the major component of this I_tail_.

Finally, we tested the effect of various Cl^−^ channel blockers on I_tail_; 100 µM NPPB, 100 µM NFA, 1 mM 4,4'-diisothiocyanatostilbene-2,2'-disulfonic acid (DIDS), and 2 mM SITS (Fig. 6B; n = 10 for each blocker). Among the extracellular application of those blockers, NPPB (90.7±10.0%) and NFA (79.8±7.3%) significantly suppressed the peak amplitude of I_tail_ without a reduction in I_Ca_. Although this I_tail_ exhibited a lower sensitivity to DIDS (62.4±12.2%) and SITS (61.2±10.8%), these results suggest that I_tail_ detected in rod bipolar cells is I_Cl(Ca)_.

### ANO1 is the CaCC that mediates I_Cl(Ca)_ in rod bipolar cells

**Figure 7:**
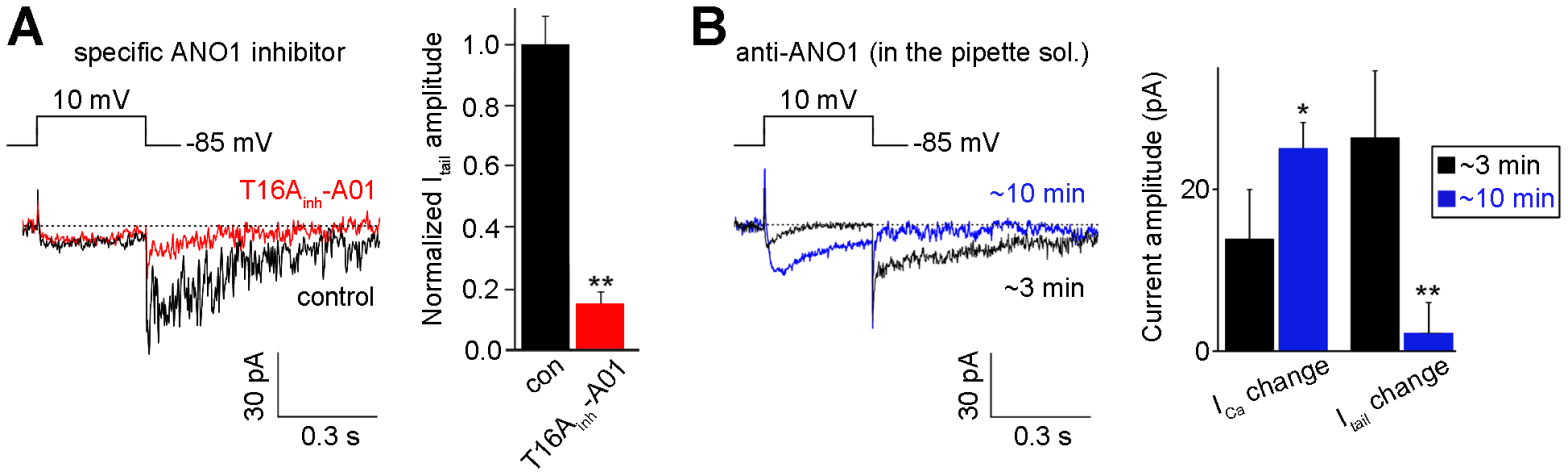
ANO1 is the molecular identity of I_Cl(Ca)_ in rod bipolar cells. **A**. The extracellular application of the specific ANO1 inhibitor T16A_inh_-A01 (10.0 µM) to the rod bipolar cell (n = 10). The results of statistical analyses are presented in the panel on the right as the normalized mean ± S.D.. **B**. Effect of the anti-ANO1 antibody on I_Cl(Ca)_ under the 20 mM [Ca^2+^]_o_ condition (n = 16). I_Ca_ and I_Cl(Ca)_ were recorded in the presence of the anti-ANO1 antibody in the pipette solution in response to depolarizing pulses ∼3 min after rupture and ∼10 min after rupture, respectively. The panel on the right depicts the comparison of the amplitude changes of I_Ca_ and I_Cl(Ca)_ between ∼3 min after rupture and ∼10 min after rupture. The results of statistical analyses are presented as the normalized mean ± S.D.. Student’s *t*-tests were used to compare the data from the 2 groups. Significance was set at *P*<0.05 (*) and *P*<0.01 (**).

We performed 2 experiments to confirm that ANO1 is the molecular identity of I_Cl(Ca)_ observed in rod bipolar cell. First, we applied a specific ANO1 inhibitor, T16A_inh_-A01 [33], which is the most recent commercially available ANO1 inhibitor. As this was the first attempt to apply T16A_inh_-A01 to retinal bipolar cells, we determined whether it acts as a specific blocker in these cells in a dose-dependent manner. The resulting dose-response curve showed that T16A_inh_-A01 inhibited I_Cl(Ca)_ in response to depolarizing voltage steps in a dose-dependent manner, with a half-maximal dose (EC_50_) of 3 µM. Upon applying the effective dose of 10 µM, an apparent inhibition of I_Cl(Ca)_ was observed without any effects on I_Ca_ (Fig. 7A; n = 12).

Next, the neutralizing effects of ANO1 were assessed using a specific antibody against ANO1, via direct addition to the pipette solution (Fig. 7B). The anti-ANO1 antibody was developed against a peptide in the first intracellular loop between transmembrane domains 2 and 3 of mouse ANO1 as a target [7], [12]. The first intracellular loop of ANO1 has recently been determined as being the critical region for voltage- and Ca^2+^-dependent gating [34]. To rule out the possibility that the anti-ANO1 effect may have arisen from the antibody itself, we tested 2 other antibodies, as negative controls: an anti-glial fibrillary acidic protein (GFAP) antibody (Fig. S4; n = 11) and a peroxidase-conjugated donkey anti-rabbit antibody (n = 11; data not shown). The application of these negative-control antibodies elicited in no significant changes in either I_Ca_ or I_Cl(Ca)_. However, the application of the anti-ANO1 antibody had a strong blocking effect on I_Cl(Ca)_ (20.1±4.1%), without changing I_Ca_ (99.5±14.6%), when current traces observed at ∼3 min were compared with those recorded at ∼10 min; the black traces were control traces recorded prior to the full diffusion of the anti-ANO1 antibody into the cell (∼3 min post-rupture), and the colored traces depicted the neutralizing effect of ANO1 via the addition of the anti-ANO1 antibody to the pipette solution (∼10 min post-rupture) (n = 12; data not shown). We further verified the blocking effect of the anti-ANO1 antibody on I_Cl(Ca)_ (14.0±6.7%) under 20 mM [Ca^2+^]_o_ conditions, which induced an increase in both I_Ca_ (146.8±11.4%) and I_Cl(Ca)_ (Fig. 7B; n = 16).

### ANO1 shortens Ca^2+^ spike-like depolarization duration in rod bipolar cells

**Figure 8:**
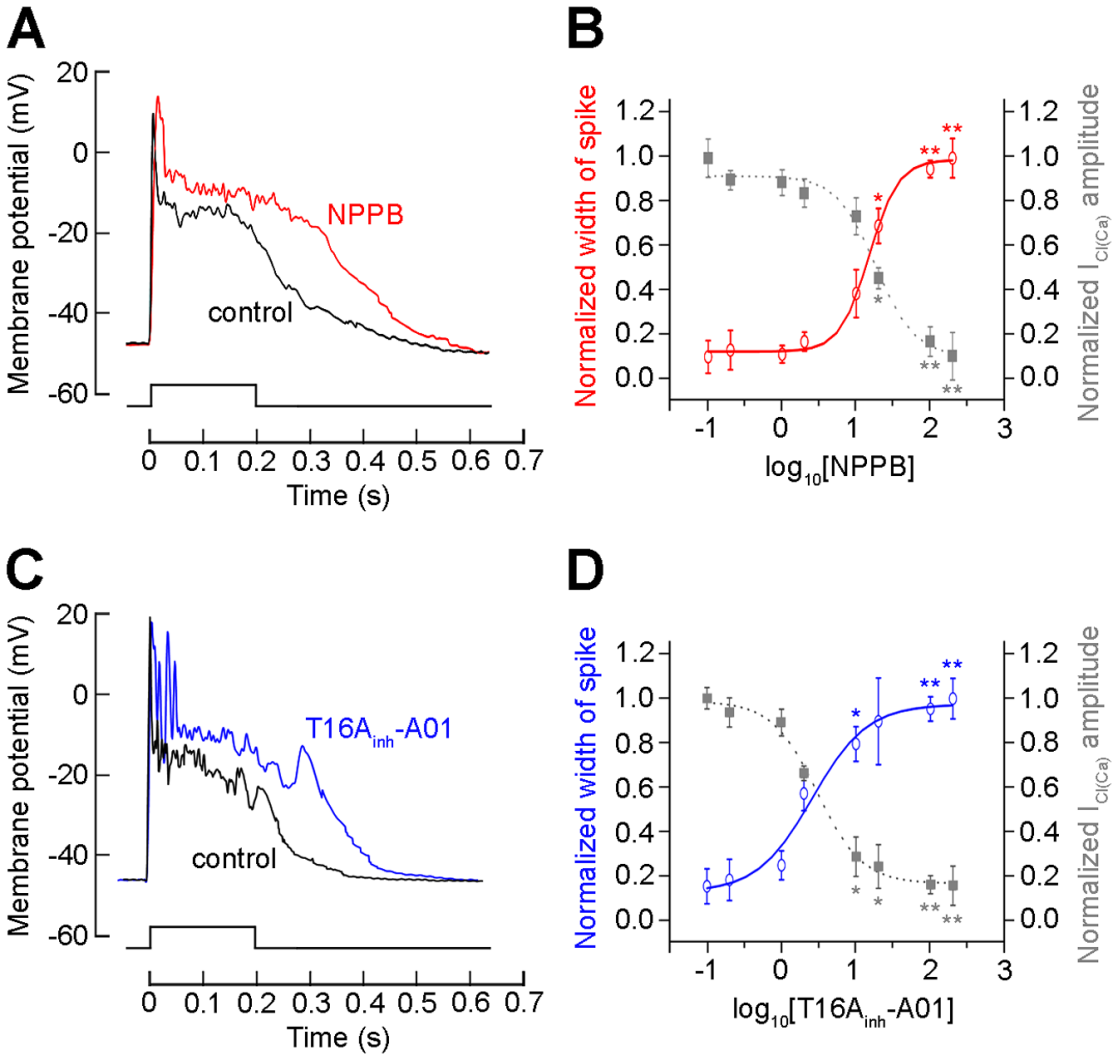
The physiological role of I_Cl(Ca)_ during membrane depolarization was examined using NPPB and T16A_inh_-A01. **A**. The bipolar cell membrane potential was adjusted to close to −45 mV with a steady hyperpolarizing current. A distinctive slow depolarizing potential that followed the Ca^2+^-dependent transient potentials was elicited by a current injection of 0.5 nA for 200 ms (n = 11). The extracellular application of 100 µM NPPB enhanced the depolarizing membrane potential compared with the control experiment. **B**. The dose-dependent decrease elicited by NPPB on I_tail_ amplitude obtained under the voltage-clamping mode (gray) was compared with the dose-dependent increase elicited by NPPB on the width of current-evoked spikes (red). The half-maximal dose (EC_50_) of NPPB that was necessary to increase the width was similar to EC_50_ of NPPB that inhibited I_Cl(Ca)_ (15.0 vs. 18.4 µM). **C**. In addition, T16A_inh_-A01 application enhanced the depolarizing membrane potential (n = 8). **D**. The dose-dependent decrease elicited by T16A_inh_-A01 on I_tail_ amplitude obtained under the voltage-clamping mode (gray) was compared with the dose-dependent increase elicited by T16A_inh_-A01 on the width of current-evoked spike (blue). The half-maximal dose (EC_50_) of T16A_inh_-A01 that was necessary to increase the width was similar to EC_50_ of T16A_inh_-A01 that inhibited I_Cl(Ca)_ (2.6 vs. 3.0 µM). Significance was set at *P*<0.05 (*) and *P*<0.01 (**).

To investigate the possible function of ANO1 on rod bipolar cells, we recorded membrane potentials using the current-clamp mode in gramicidin-perforated whole cells. It has been demonstrated that rod bipolar cells dissociated from the mammalian retina exhibit membrane potentials of about −45 mV [35]–[37] and E_Cl_ measured in rod bipolar cell axon terminals is about −60 mV [38]. The application of depolarizing currents (0.05∼0.1 nA) to cells resting near −45 mV (n = 14) led to the observation of a distinct Ca^2+^ spike-like depolarization in isolated rod bipolar cells (Figs. 8A and C). This waveform consisted of a first few huge spikes and subsequent small spikes riding atop a plateau potential that was similar to an excitatory postsynaptic potential. The application of 100 µM NPPB and T16A_inh_-A01 to the bath (Figs. 8A and C; n = 11 for each) revealed that the very first spike was maintained, whereas the depolarizing potential waveform was prolonged. The measurement of the width of the waveform at 1/2 total spike height showed that the application of NPPB and T16A_inh_-A01 augmented the width of current-evoked response potential by ∼60% compared with that observed in the absence of the drugs (Figs. 8B and D). Interestingly, EC_50_ of in increasing the width was similar to EC_50_ of the 2 Cl^−^ channel blockers in inhibiting I_Cl(Ca)_ (Figs. 8B and D; NPPB, 15.0 vs. 18.4 µM; T16A_inh_-A01, 2.6 vs. 3.0 µM), indicating a close relationship between I_Cl(Ca)_ and current-evoked depolarization. These results suggest that ANO1 contributes to the shaping of the spike waveform in rod bipolar cells.

## Discussion

This study documented the expression of ANO1, a CaCC, in the retina. This was the first demonstration of the presence of I_Cl(Ca)_ in rod bipolar cells, which are a specific type of retinal neurons that express ANO1, and its possible function. Our results showed that (1) various retinal neurons expressed ANO1; (2) this protein was exclusively localized in the presynaptic region; (3) rod bipolar cells expressing ANO1 conferred I_Cl(Ca)_, which was blocked by T16A_inh_-A01 (a selective ANO1 inhibitor) and a neutralizing antibody; and (4) NPPB and T16A_inh_-A01 prolonged the distinct Ca^2+^ spike-like depolarization evoked by current injection in dissociated rod bipolar cells. These results suggest that ANO1 acts as an intrinsic regulator of the presynaptic membrane potential during synaptic transmission in retinal neurons.

In sensory neurons, CaCCs are localized in somatodendritic regions and are involved in signal transduction and amplification, ANO1 activation leads to depolarization in DRG neurons and acts as a heat sensor [16], whereas ANO2 contributes to signal amplification of olfactory sense in ORNs [39], [40]. In addition, Huang et al. [41] have demonstrated that ANO2 is localized in the close vicinity of voltage-gated Ca^2+^ channels and NMDA receptors in the somatodendritic region in hippocampal neurons, where it regulates action potentials and synaptic responses. Thus, CaCCs appear to play important functions in receptive and/or postsynaptic regions in neurons.

We showed that ANO1 was strongly expressed in 2 synaptic layers. Double-labeling experiments using various pre- and post-synaptic markers and immunoelectron microscopy clearly showed that ANO1 was localized to presynaptic terminals such as photoreceptor terminals, bipolar axon terminals, and amacrine cell processes. This preferential presynaptic localization of ANO1 was also observed in the cochlea, where ANO1 is exclusively localized at medial olivocochlear efferent nerve endings [42], and in the cerebellum where ANO1 is mainly found in mossy fibers (our unpublished data). Furthermore, when we performed whole-cell voltage clamp recordings on a rod bipolar cell without the axon terminal, which is often lost during the procedure of enzymatic dissociation, I_Cl(Ca)_ was hardly elicited (Fig. S5), suggesting that I_Cl(Ca)_ may originate from the axon terminal and/or be linked with the Ca^2+^ channels localized at the axon terminal. These findings suggest that CaCCs also have important functions in presynaptic terminals in neurons. In fact, the exclusive presynaptic localization of ANO1 in photoreceptor terminals observed in this study concurs with previous reports that showed that I_Cl(Ca)_ is elicited by depolarization-evoked Ca^2+^ influx, which activates CaCCs localized at photoreceptor terminals [17], [18], [25], [38], and supports the proposed role of I_Cl(Ca)_ in membrane potential stabilization during synaptic activity [19], [20], [22], [43] and presynaptic Ca^2+^ channel modulation [23]–[25].

In this study, the abundant expression of ANO1 detected in various retinal neurons, such as photoreceptors and bipolar and amacrine cells, exceeded our expectations. For example, the presence of I_Cl(Ca)_ in mouse rod bipolar cells has not been described previously, even though many studies on Ca^2+^ channel properties [30], [31] and Ca^2+^ tail currents [44], [45] have been performed to understand visual signal processing and synaptic transmission in mammalian rod bipolar cells. The reasons for this may be that I_Ca_ amplitude that causes I_Cl(Ca)_ is too small in mouse rod bipolar cells and that the studies mentioned above focused on the detection and characterization of I_Ca_ itself. In this study, we found that ANO1 was expressed in rod bipolar cells; hence, we anticipated the presence of I_Cl(Ca)_ in these cells. To facilitate the identification of I_Cl(Ca)_, we lowered the concentration of the Ca^2+^ chelator (0.5 mM EGTA) compared with that used in previous studies (5 mM EGTA) [30], [31], [46]. In these conditions, we were able to identify I_Cl(Ca)_ in rod bipolar cells easily and successfully. In fact, using this protocol (0.5 mM EGTA), I_Cl(Ca)_ was identified in goldfish retinal bipolar cells [28] after I_Ca_ identification [46], [47]. Thus, to detect the presence and identify the function of CaCCs that are underestimated in the nervous system, further studies aimed at identifying CaCC conductance in other central neurons by using our protocol are needed.

Here, the suppression of CaCC using NPPB, which is a CaCC blocker, and T16A_inh_-A01, which is a selective ANO1 blocker, prolonged the current-evoked depolarization that was experimentally induced by us. These results provide a very critical piece of information that explains the possible roles of I_Cl(Ca)_ mediated by ANO1 in rod bipolar cells. The dissociated preparation can be considered as a condition in which the synaptic network is free (i.e., synaptic events do not occur between presynaptic and postsynaptic neurons), but in which the intrinsic properties of the recording cells are preserved. The function of CaCCs may depend on their spatial distribution in cells and on local differences in E_Cl_. Notably, to establish the function of I_Cl(Ca)_, it is crucial to know E_Cl_. CaCCs promote membrane depolarization in rod photoreceptor terminals, where E_Cl_ is ≈−20 mV and the membrane potential is estimated at ∼−45 mV [48]. Conversely, mouse rod bipolar cells, which receive synaptic inputs from rods, have E_Cl_ (≈−60 mV) that is more negative than the resting membrane potential (≈−45 mV) at its presynaptic axon terminal [38], [49]. Under such conditions, activation of Ca^2+^ channels may open ANO1 in rod bipolar cells resulting in an outward current that shortens Ca^2+^ spike-like depolarizations or facilitates the repolarization of the cell membrane. Taken together, these results suggest that the physiologic function of ANO1 is dependent on Cl^−^ distribution and the establishment of E_Cl_. ANO1 appears to confer an intrinsic electrical characteristic to retinal neurons and in the same way, may act as an important intrinsic regulator of the membrane potential in central neurons, including retinal neurons.

In addition, during synaptic transmission in rod bipolar axon terminals in which E_Cl_ is more negative than the resting membrane potential, Cl^−^ conductance stabilizes the presynaptic membrane potential, allowing an increase in glutamate release via an increase in [Ca^2+^]_i_ [19], [24]. Vesicle release is regulated by L-type Ca^2+^ channels, which in turn are regulated by Cl^−^ moving through CaCCs, as reported at photoreceptor ribbon synapses [17], [18], [25], [38]. AII amacrine cells, which are located postsynaptically rod bipolar cells exhibit a mixture of transient and sustained components [50]–[52]. The transient component of the postsynaptic current is quite pronounced, whereas the sustained component is of relatively low amplitude [53]–[55]. This pattern is observed even in the presence of blockers of inhibitory GABAergic input and when only sustained Ca^2+^ currents are activated in the rod bipolar cell, which suggests that glutamate release from mammalian rod bipolar cells is inherently transient [53], [56], [57]. In this study, a CaCC blocker and a selective ANO1 blocker prolonged the current-evoked depolarization in an isolated cell preparation without the synaptic network, suggesting that ANO1 function may be attributed to inherently transient glutamate release in rod bipolar cells.

## Materials and Methods

### Ethical standards

This study was carried out in strict accordance with the recommendations provided in the Guide for the Care and Use of Laboratory Animals of the National Institutes of Health (NIH Publications No. 80-23; revised in 1996). The study protocol was approved by the Institutional Animal Care and Use Committee (IACUC) of the College of Medicine, The Catholic University of Korea (Approval Number: CUMS-2012-0087-01). All animal surgeries were performed under ketamine and xylazine anesthesia, and all efforts were made to minimize suffering.

### Animals and tissue preparation

Three-month-old male C57BL/6 mice (Orient Bio, Seongnam, Korea) were used in this study. The mice were euthanized with 15% chloral hydrate.

For western blot analysis, the animals were transcardially perfused with saline, the eyeballs were enucleated, the anterior segments of the eyeballs were removed, and the retinal tissues were quickly dissected on an ice-cold plate, frozen on dry ice, and stored at −70°C.

For immunohistochemistry, the eyecups were fixed by immersion in 4% paraformaldehyde in 0.1 M phosphate buffer (PB, pH 7.4). After fixation, the retinas were carefully dissected and transferred to 30% sucrose in 0.1 M PB. They were then frozen in liquid nitrogen, thawed, and stored at −70°C.

For patch-clamp recordings, the retinas were quickly dissected and treated with a low-Ca^2+^ solution containing 4 mg/mL of papain activated by 10 mM L110 cysteine. Subsequently, retinal cells were enzymatically isolated from retinas. The dissociated retinal preparations were kept in an oxygenated chamber during the recordings.

### Antibodies

An anti-ANO1 polyclonal antibody (Cat. #LF-PA0208; Ab Frontier, Seoul, Korea) was raised in rabbits against a synthetic peptide with of amino acid sequence KDHPRAEYEARVLEKS (amino acids 451–466), which corresponded to a stretch located in the first intracellular loop between transmembrane domains 2 and 3 of mouse ANO1. The specificity of this antibody was tested by western blotting and immunocytochemical analyses in our previous study [7], [42] and demonstrated by immunohistochemistry in knockout animals [12].

An anti-glial fibrillary acidic protein (GFAP) (Millipore, Temecula, CA) and peroxidase-conjugated donkey anti-rabbit (Molecular Probes, Eugene, OR) antibodies were used as controls in the patch-clamp recording experiment that was performed to test the neutralizing effect of the anti-ANO1 antibody. For double-labeling, various antibodies were used to label presynaptic or postsynaptic elements and specific neurons in the retina. Their target structures, together with dilution rates, sources, companies, and references, are listed in Table S1.

### Western blotting

Western blot analysis was performed on extracts of the prepared tissues, which were homogenized in ice-cold RIPA buffer (50 mM Tris buffer, pH 8.0; 150 mM NaCl; 1% NP-40; 0.5% deoxycholate; and 0.1% SDS). Samples from each tissue (corresponding to 50 µg of total protein) were separated by SDS-PAGE, and the proteins were blotted onto a nitrocellulose membrane and probed with the anti-ANO1 antibody (dilution, 1∶2000). The immunoreactive bands were detected using an Enhanced Chemiluminescence Detection Kit (Amersham, Arlington Heights, IL). Preincubation of the anti-ANO1 antibody with a 10-fold excess (w/w) of the antigenic peptide led to the absence of labeling in the subsequent western blot analysis (Fig. 1A); moreover, the reaction of the western blots with the secondary antibody alone produced no signal (data not shown).

### Immunohistochemistry

Fifty-micrometer-thick vertical vibratome sections for the retina were used for immunohistochemistry. The sections were incubated in 10% normal donkey serum and incubated with the polyclonal antibody against ANO1 (dilution, 1∶500) in 0.01 M phosphate-buffered saline (PBS, pH 7.4) containing 0.5% Triton X-100 for 1 day at 4°C. The sections were washed in PBS and incubated in the presence of biotin-labeled donkey anti-rabbit IgG (dilution, 1:100; Jackson Immuno Research, West Grove, PA) for 2 h. Subsequently, the sections were washed and incubated with Cy3-conjugated streptavidin in PBS (dilution, 1:1000; Jackson Immuno Research) for 1 h. The fluorescent specimens were mounted using Vectashield mounting medium (Vector Laboratories, Burlingame, CA). The specificity of the immunostaining was evaluated in retinal sections by preincubating the anti-ANO1 antibody with a 10-fold excess of its antigenic peptide for 1 h at room temperature (Fig. 1B) and by omitting the incubation step with the primary antibody (data not shown); no staining was observed in these sections.

For double-labeling experiments, the sections were incubated with antibody mixtures composed of the anti-ANO1 antibody and one of the marker antibodies, followed by incubation in the presence of appropriate secondary antibodies conjugated with Cy3 (Jackson Immuno Research) and Alexa Fluor 488 (Molecular Probes) at a dilution of 1:200.

Digital images (1,024×1,024 pixels) were acquired using a Zeiss LSM 510 Meta confocal microscope (Carl Zeiss Co. Ltd., Germany) and were imported into Photoshop (Adobe Systems, San Jose, CA). The brightness and contrast of the final images were adjusted.

### Immunoelectron microscopy

Retinal sections were prepared as described above. After blocking, the sections were incubated in a solution containing the anti-ANO1 antibody at 4°C for 1 day, as described for light microscopy but without Triton X-100. The sections were washed in PBS for 45 min (3×15 min), incubated with biotin-labeled donkey anti-rabbit IgG (dilution, 1∶100; Jackson Immuno Research) for 2 h, and washed 3 times in PBS for 45 min (3×15 min). The sections were then incubated in an avidin-biotin-peroxidase complex (ABC) solution (Vector Laboratories) for 1 h, washed in 0.1 M Tris buffer (TB, pH 7.6), and preincubated in 0.05% 3,3'-diaminobenzidine tetrahydrochloride (DAB) in TB for 10 min, followed by incubation in the same solution containing 0.05% hydrogen peroxide (H_2_O_2_) for an additional 10 min. The reaction was monitored using a low-power microscope and was stopped by replacing the DAB and H_2_O_2_ solution with TB.

Stained sections were postfixed in 1% glutaraldehyde in PB for 1 h. After washing in PB containing 4.5% sucrose for 15 min (3×5 min), the sections were postfixed in 1% OsO_4_ in PB for 1 h. Subsequently, the sections were rewashed in PB containing 4.5% sucrose and dehydrated in a graded series of ethanol. During the dehydration procedure, the sections were stained *en bloc* with 1% uranyl acetate in 70% alcohol for 1 h, then transferred to propylene oxide, and flat embedded in Epon 812. After curing at 60°C for 3 days, well-stained areas were cut out and attached to an Epon support for further ultrathin sectioning (Reichert-Jung, Nuβloch, Germany). Serial ultrathin sections (70–90 nm in thickness) were collected on single slot grids, and examined using a JEM 1010 electron microscope (JEOL, Tokyo, Japan).

### Patch-clamp recordings

Whole-cell recordings were performed on rod bipolar cells by using an EPC-9 amplifier and the PULSE software (Heka Electronik, Germany). Electrodes were fabricated from borosilicate microcapillary tubes (VWR Scientific, West Chester, PA) and fire-polished. The resistance of the electrode was 8–12 MΩ. Series resistance ranged from 25 to 40 MΩ. The extracellular solution contained (in mM): NaCl, 135; CsCl, 5; CaCl_2_, 10; MgCl_2_, 1; glucose, 10; and HEPES, 10. The pipette solution used for voltage-clamp recordings contained (in mM): CsCl, 120; MgCl_2_, 1; CaCl_2_, 0.5; EGTA, 0.5; tetraethylammonium (TEA)-Cl, 20; and Tris-ATP, 10 (pH adjusted to 7.2 with CsOH). The pipette solution used for current-clamp recordings in the perforated patch-clamp mode was (in mM): KCl, 140; MgCl_2_, 1; CaCl_2_, 0.5; EGTA, 0.5 and Tris-ATP, 10 and gramicidine D (14.8 µg/mL) (pH adjusted to 7.2 with KOH). Dissociated rod bipolar cells were used in recordings for 4–5 h after isolation.

Current signals were filtered at 3 kHz and digitized at 5 kHz by using a data-acquisition interface (LIH 1600 A/D board, HEKA). Cell membrane capacitance and series resistance current were automatically compensated by the amplifier using the software. The calculated liquid junction potential was also subtracted using this software. Data were analyzed offline by using the PULSE-fit and ORIGIN programs. Statistical analysis was performed using analysis of variance (ANOVA) and Student’s *t* tests. Data are represented as means ± S.D.. The differences were considered significant at *P*<0.05.

### Pharmacology

Test solutions were mostly dissolved in the Ringer solution and applied locally to a whole-cell clamped cell from a puffer pipette via Y-tube system. All chemicals were purchased from Sigma-Aldrich (St Louis, MO), with the exception of 2-(5-ethyl-4-hydroxy-6-methylpyrimidin-2-ylthio)-N-(4-(4-methoxyphenyl)thiazol-2-yl)acetamide (T16A_inh_-A01) (Tocris, Ellisville, MI), dl-threo-β-benzyloxyaspartate (dl-tBOA) (Tocris), the anti-ANO1 antibody (Ab Frontier), and the anti-GFAP antibody (Millipore).

Some pharmacological agents were used for the manipulation of a specific ionic current: CoCl_2_ for blocking Ca^2+^ current and CsCl and TEA in the pipette solution for blocking h-current and voltage- and Ca^2+^-activated K^+^ currents. Nifedipine was used to block an L-type Ca^2+^ channel. The Ca^2+^ chelators 1,2-bis [o-aminophenoxy]ethane-N,N,N′,N′-tetraacetic acid (BAPTA) and ethylene glycol-bis [2-aminoethylether]-*N,N,N′,N′*-tetraacetic acid (EGTA), were applied in the pipette solution; when these agents were applied at higher concentration, the concentration of CsCl was appropriately reduced to adjust the osmolarity. 1,2-bis [2-aminophenoxy]ethane-N,N,N′,N′-tetraacetic acid tetrakis (BAPTA/AM), which is a cell-permeable Ca^2+^ chelator, was applied extracellularly at 0.1 mM. To suppress Cl^−^ conductance, several agents were applied, i.e., 5-nitro-2-(3-phenylpropylamino) benzoic acid (NPPB), niflumic acid (NFA), 4,4'-diisothiocyanatostilbene-2,2'-disulfonic acid (DIDS), and 4-acetamido-4'-isothiocyanatostilbene-2,2'-disulfonic acid (SITS), to block Cl^−^ channels selectively. T16A_inh_-A01 was tested as a specific ANO1 inhibitor. dl-tBOA was used to suppress Cl^−^ current mediated by the glutamate transporter. In addition, we tested the effects of blocking ANO1 by adding the specific anti-ANO1 antibody directly to the pipette solution. Both the anti-ANO1 and anti-GFAP antibodies were used at dilutions of 1:500 in the pipette solution.

## Supporting Information

Figure S1 **Cellular and subcellular localization of ANO1 in the mouse retina.** Confocal micrographs taken from vertical vibratome sections (50 µm in thickness) processed for double labeling with antibodies against ANO1 (red) and Goα (**A**, green) or calbindin (**B**, green) or SMI32 (**C**, green) in the mouse retina. (**A, B**) Large and small ANO1-labeled puncta are visible in the OPL. In **A**, the anti-Goα antibody labels the somata of ON-bipolar cells (asterisks) in the outer INL and their dendrites extending into the OPL. In the merged image, ANO1 and Goα are not colocalized. In **B**, 2 calbindin-labeled horizontal cell somata (asterisks) and their dendrites located in the inner OPL are seen. In the merged image, ANO1-positive cells do not show calbindin immunoreactivity. **C**. Numerous ANO1-labeled puncta of various sizes are observed in the IPL. A SMI32-labeled ganglion cell soma (asterisk) and labeled dendrites are seen in the GCL and IPL, respectively. In the merged image, ANO1-immunoreactive puncta are not localized to SMI32-labeled ganglion cell dendrites in the IPL. Scale bars, 20 µm.
(TIF)

Figure S2 **Blocking effect of nifedipine on L-type Ca^2+^ channels in rod bipolar cells.** Nifedipine (10 µM) was applied to the rod bipolar cell. Nifedipine decreased the sustained component of I_Ca_ and I_tail_ (n = 14). The results of statistical analyses are presented in the panel on the right as the normalized mean ± S.D.. Student’s *t* tests were used to compare the data from the 2 groups.
(TIF)

Figure S3 **Dependency of** **I_tail_ on [Ca^2+^]_i_.** The dependency of I_tail_ on [Ca^2+^]_i_ was confirmed via the application of 2 Ca^2+^ chelators in the pipette solution. The introduction of both BAPTA (>10 mM) (n = 8) and EGTA (5 mM) (n = 8) into bipolar cells via a recording pipette strongly suppressed I_tail_. The results of statistical analyses are presented in the panel on the right as the normalized mean ± S.D.. Student’s *t* tests were used to compare the data from the 2 groups. Significance was set at *P*<0.01 (**) and *P*<0.001 (***).
(TIF)

Figure S4 **A negative-control experiment of the blocking effect of the neutralizing antibody on I_Cl(Ca)_.** A GFAP-conjugated donkey anti-rabbit antibody was introduced directly in the pipette solution to examine the blocking effect of the neutralizing antibody on I_Cl(Ca)_. In the presence of the anti-GFAP antibody, both I_Ca_ and I_Cl(Ca)_ were recorded at a holding potential of −85 mV in response to depolarizing pulses of +10 mV ∼3 min after rupture and ∼10 min after rupture (n = 11). The panel on the right depicts the comparison of the amplitude changes of I_Ca_ and I_Cl(Ca)_ between ∼3 min after rupture and ∼10 min after rupture. Student’s *t* tests were used to compare the data from the 2 groups.
(TIF)

Figure S5 **I_tail_ is absent in bipolar cells without axon terminals**. A dissociated rod bipolar cell was filled with Lucifer Yellow during recording and was morphologically identified under a fluorescence microscope after recording (right panel). The representative trace recorded from a rod bipolar cell without axon terminals showed the presence of I_Ca_ and the absence of I_tail_. The currents were recorded at the voltage of +10 mV from a holding potential of −85 mV. Scale bar, 5 µm.
(TIF)

Table S1 **Immunologic marker antibodies used in this study.**
(DOC)
