# Supplementary figures and images for: Presynaptic Localization and Possible Function of Calcium-Activated Chloride Channel Anoctamin 1 in the Mammalian Retina
Source: PLoS One. 2013 Jun 26;8(6):e67989. doi: 10.1371/journal.pone.0067989 (PMC3693959; doi:10.1371/journal.pone.0067989)

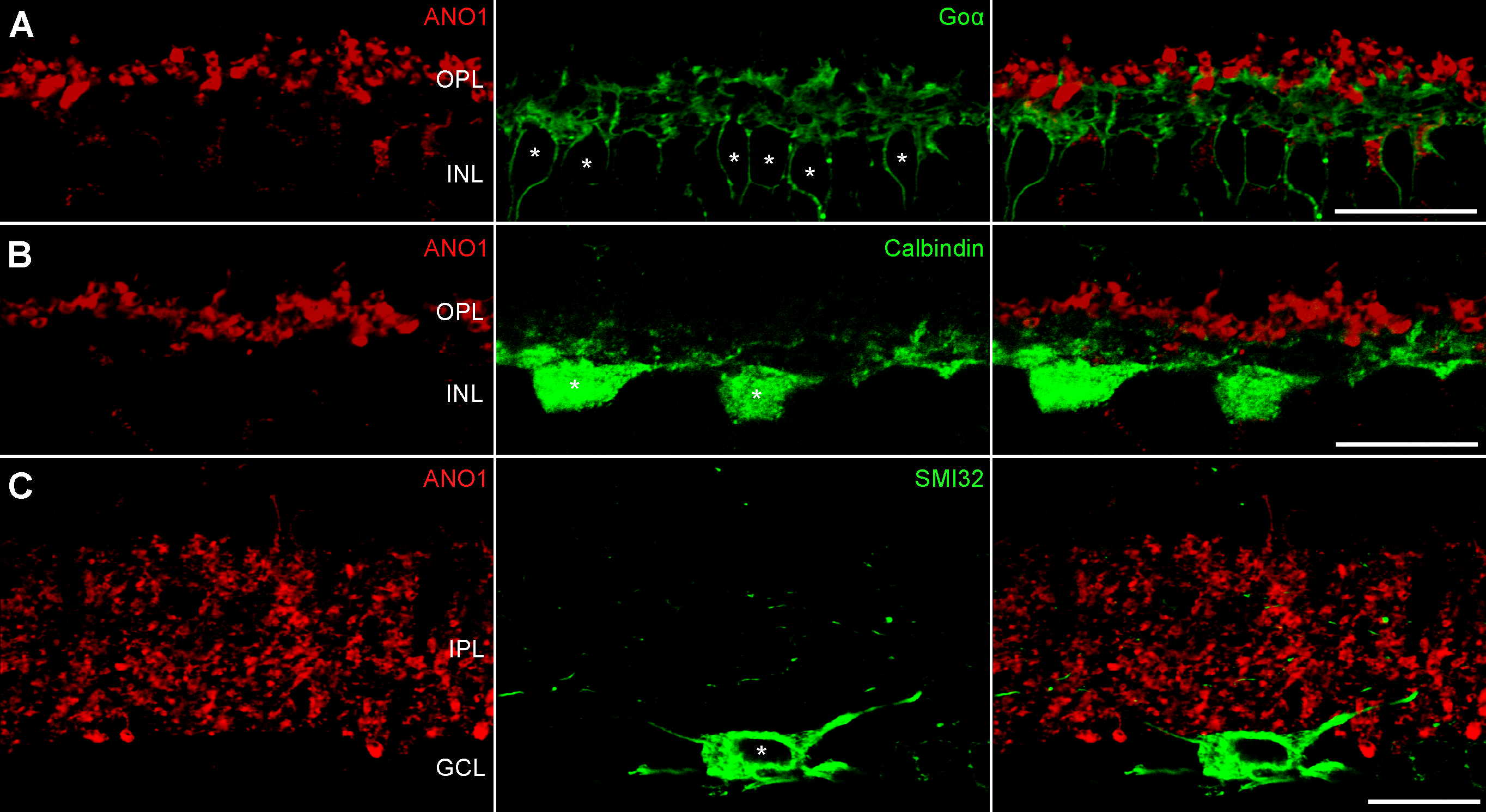

Supplement: Figure S1 — Cellular and subcellular localization of ANO1 in the mouse retina. Confocal micrographs taken from vertical vibratome sections (50 µm in thickness) processed for double labeling with antibodies against ANO1 (red) and Goα (A, green) or calbindin (B, green) or SMI32 (C, green) in the mouse retina. (A, B) Large and small ANO1-labeled puncta are visible in the OPL. In A, the anti-Goα antibody labels the somata of ON-bipolar cells (asterisks) in the outer INL and their dendrites extending into the OPL. In the merged image, ANO1 and Goα are not colocalized. In B, 2 calbindin-labeled horizontal cell somata (asterisks) and their dendrites located in the inner OPL are seen. In the merged image, ANO1-positive cells do not show calbindin immunoreactivity. C. Numerous ANO1-labeled puncta of various sizes are observed in the IPL. A SMI32-labeled ganglion cell soma (asterisk) and labeled dendrites are seen in the GCL and IPL, respectively. In the merged image, ANO1-immunoreactive puncta are not localized to SMI32-labeled ganglion cell dendrites in the IPL. Scale bars, 20 µm. (TIF) [file pone.0067989.s001.tif]

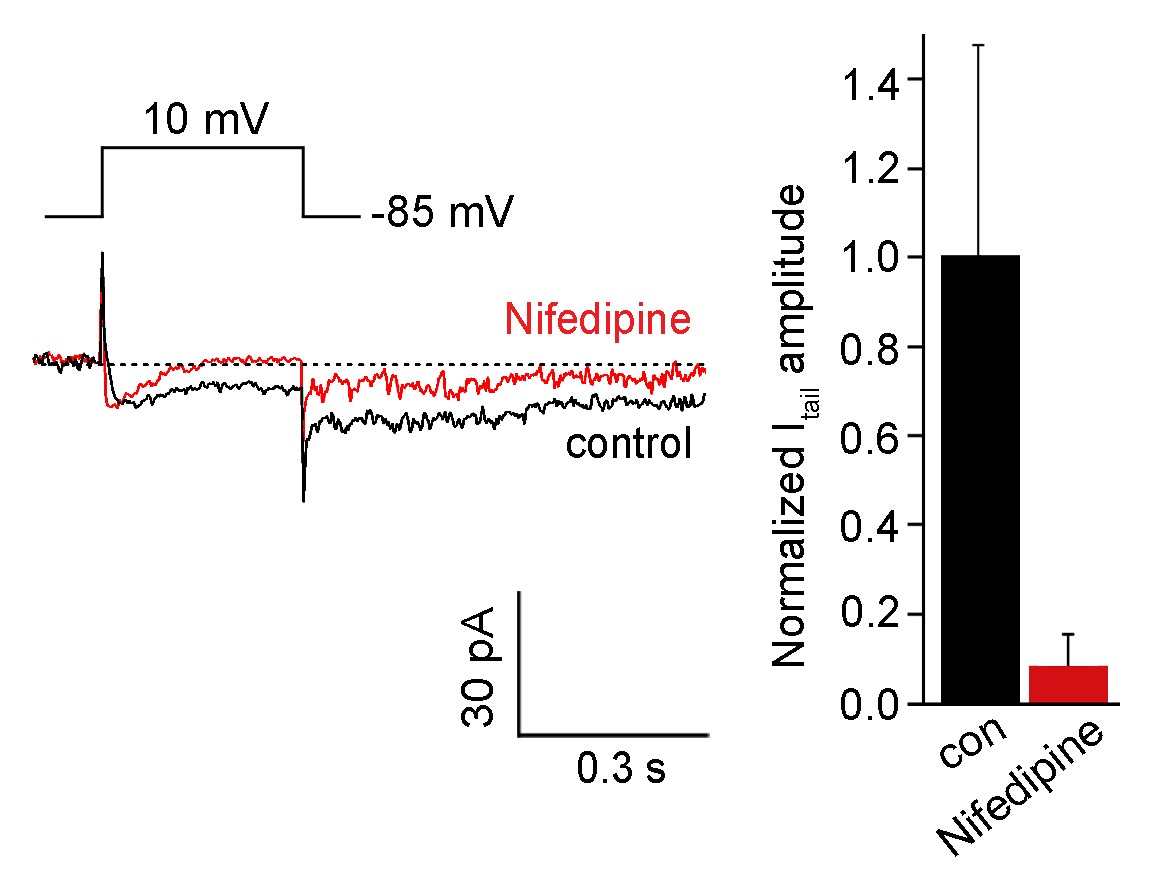

Supplement: Figure S2 — Blocking effect of nifedipine on L-type Ca2+ channels in rod bipolar cells. Nifedipine (10 µM) was applied to the rod bipolar cell. Nifedipine decreased the sustained component of ICa and Itail (n = 14). The results of statistical analyses are presented in the panel on the right as the normalized mean ± S.D.. Student’s t tests were used to compare the data from the 2 groups. (TIF) [file pone.0067989.s002.tif]

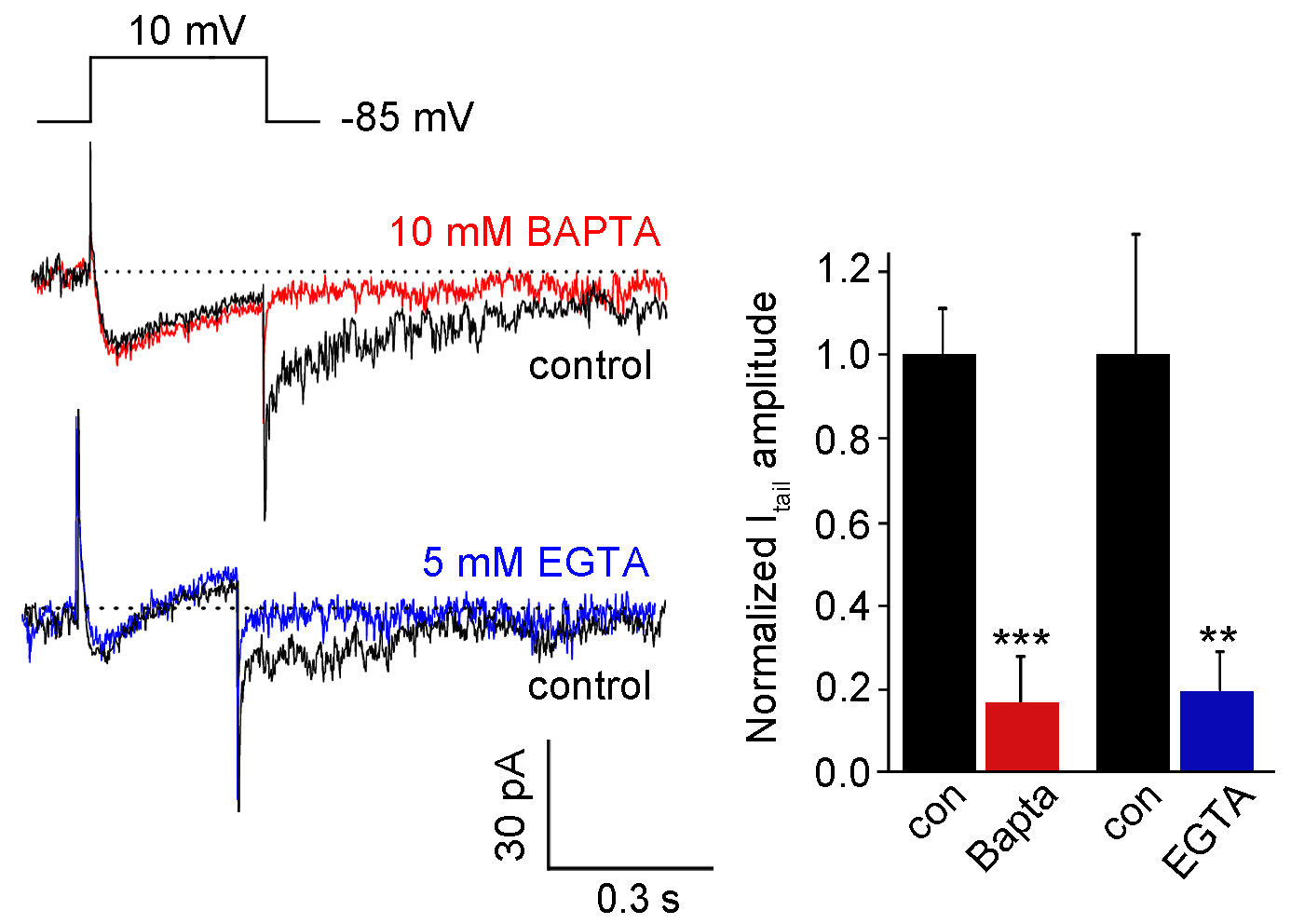

Supplement: Figure S3 — Dependency of Itail on [Ca2+]i. The dependency of Itail on [Ca2+]i was confirmed via the application of 2 Ca2+ chelators in the pipette solution. The introduction of both BAPTA (>10 mM) (n = 8) and EGTA (5 mM) (n = 8) into bipolar cells via a recording pipette strongly suppressed Itail. The results of statistical analyses are presented in the panel on the right as the normalized mean ± S.D.. Student’s t tests were used to compare the data from the 2 groups. Significance was set at P<0.01 (**) and P<0.001 (***). (TIF) [file pone.0067989.s003.tif]

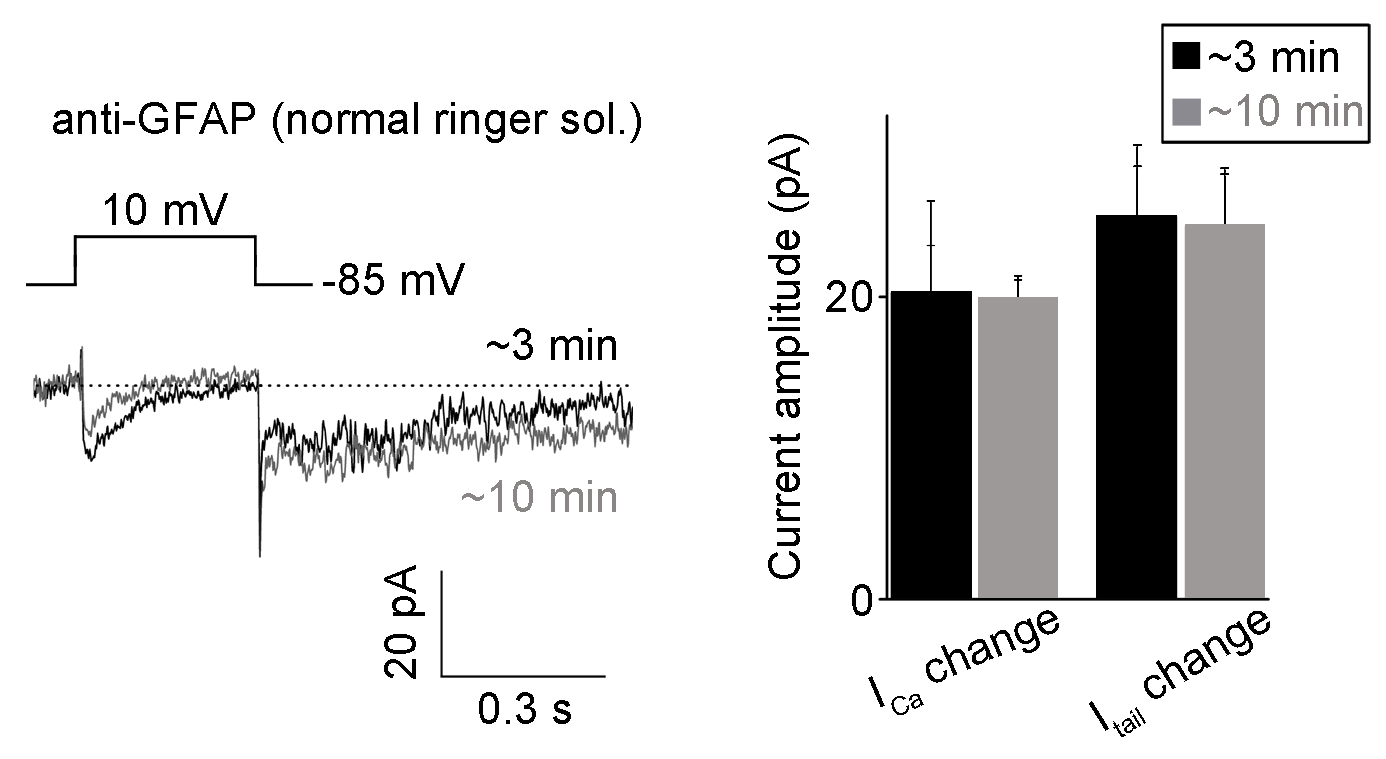

Supplement: Figure S4 — A negative-control experiment of the blocking effect of the neutralizing antibody on ICl(Ca). A GFAP-conjugated donkey anti-rabbit antibody was introduced directly in the pipette solution to examine the blocking effect of the neutralizing antibody on ICl(Ca). In the presence of the anti-GFAP antibody, both ICa and ICl(Ca) were recorded at a holding potential of −85 mV in response to depolarizing pulses of +10 mV ∼3 min after rupture and ∼10 min after rupture (n = 11). The panel on the right depicts the comparison of the amplitude changes of ICa and ICl(Ca) between ∼3 min after rupture and ∼10 min after rupture. Student’s t tests were used to compare the data from the 2 groups. (TIF) [file pone.0067989.s004.tif]

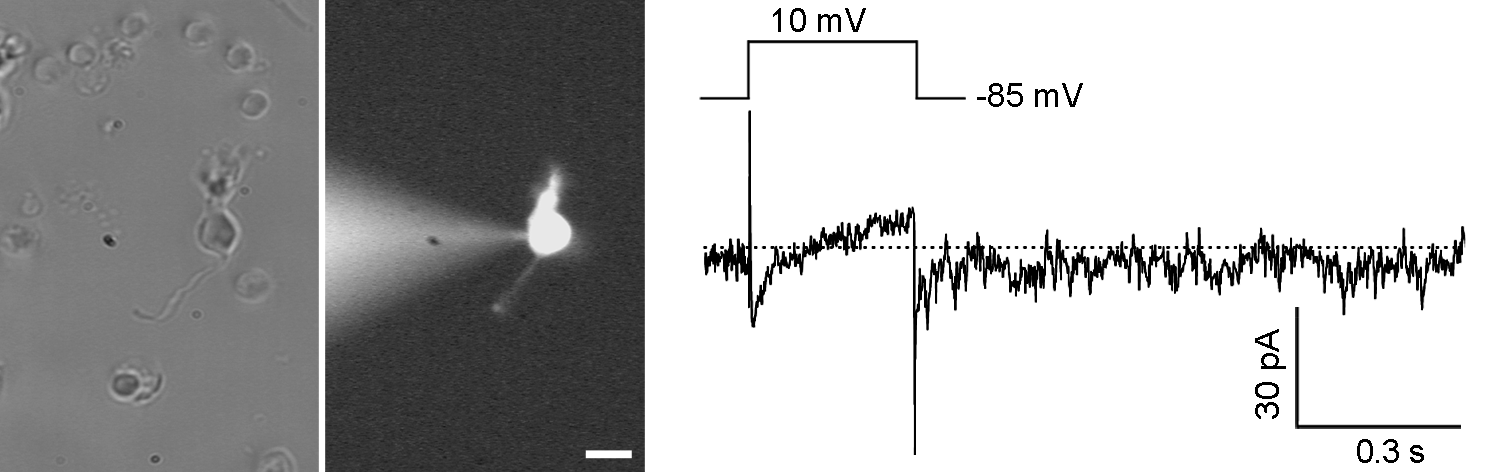

Supplement: Figure S5 — Itail is absent in bipolar cells without axon terminals. A dissociated rod bipolar cell was filled with Lucifer Yellow during recording and was morphologically identified under a fluorescence microscope after recording (right panel). The representative trace recorded from a rod bipolar cell without axon terminals showed the presence of ICa and the absence of Itail. The currents were recorded at the voltage of +10 mV from a holding potential of −85 mV. Scale bar, 5 µm. (TIF) [file pone.0067989.s005.tif]
