# Supplementary material for: Presynaptic Localization and Possible Function of Calcium-Activated Chloride Channel Anoctamin 1 in the Mammalian Retina
Source: PLoS One. 2013 Jun 26;8(6):e67989. doi: 10.1371/journal.pone.0067989 (PMC3693959; doi:10.1371/journal.pone.0067989)
Supplement: Table S1 — Immunologic marker antibodies used in this study. (DOC) [file pone.0067989.s006.doc]

**Table S1. Immunological marker antibodies used in this study**

| Antibody | Target structure | Dilution rate | Source | Company (Cat. No.) | References |
| --- | --- | --- | --- | --- | --- |
| Anti-ANO1 | ANO1 | 1:1000 | Rabbit | AB Frontier (LF-PA0208) | Yang et al., 2008  Romanenko et al., 2010 |
| Anti-synaptophysin | Photoreceptor terminals | 1:500 | Mouse | Sigma  (S5768) | Cuenca et al., 2005  Sullivan et al., 2007 |
| Anti-VGLUT1 | Photoreceptor terminals  Bipolar cell axon terminals | 1:1000 | Guinea pig | Chemicon  (AB5905) | Gong et al., 2006  Puthussery et al., 2011 |
| Anti-calbindin | Horizontal cells | 1:1000 | Mouse | Sigma  (C9848) | O’Brien et al., 2006  Puthussery et al., 2011 |
| Anti- Goα | ON-bipolar cell dendrites | 1:100 | Mouse | Chemicon  (MAB3073) | Huang et al., 2003  Koike et al., 2010 |
| Anti-VGAT | Amacrine cell processes | 1:500 | Mouse | Synaptic Systems  (131 011) | Witkovsky et al., 2008  Guo et al., 2009 |
| Anti-SMI32 | Ganglion cell dendrites | 1:500 | Mouse | Stemberger  (SMI32-R) | Coombs et al., 2006  Lim et al., 2007 |
| Anti-PKC | Rod bipolar cells | 1:500 | Mouse | Santa Cruz  (sc-80) | Johnson et al., 2003  Zahir et al., 2005 |
| Anti-GFAP | Müller cell | 1:1000 | Rabbit | Millipore  (AB5804) | Barcelona et al., 2011  Xue et al., 2011 |

**References in Table S1**

1. Yang YD, Cho H, Koo JY, Tak MH, Cho Y, et al. (2008) TMEM16A confers receptor-activated calcium-dependent chloride conductance. Nature 455: 1210-1215.

2. Romanenko VG, Catalán MA, Brown DA, Putzier I, Hartzell HC, et al. (2010) Tmem16A encodes the Ca^2+^-activated Cl^-^ channel in mouse submandibular salivary gland acinar cells. J Biol Chem 285: 12990-13001.

3. Cuenca N, Pinilla I, Sauvé Y, Lund R (2005) Early changes in synaptic connectivity following progressive photoreceptor degeneration in RCS rats. Eur J Neurosci 22: 1057-1072.

4. Sullivan RK, Woldemussie E, Pow DV (2007) Dendritic and synaptic plasticity of neurons in the human age-related macular degeneration retina. Invest Ophthalmol Vis Sci 48: 2782-2791.

5. Gong J, Jellali A, Mutterer J, Sahel JA, Rendon A, et al. (2006) Distribution of vesicular glutamate transporters in rat and human retina. Brain Res 1082: 73-85.

6. Puthussery T, Gayet-Primo J, Taylor WR, Haverkamp S (2011) Immunohistochemical identification and synaptic inputs to the diffuse bipolar cell type DB1 in macaque retina. J Comp Neurol 519: Spc1.

7. O'Brien JJ, Li W, Pan F, Keung J, O'Brien J, et al. (2006) Coupling between A-type horizontal cells is mediated by connexin 50 gap junctions in the rabbit retina. J Neurosci 26: 11624-11636.

8. Huang L, Max M, Margolskee RF, Su H, Masland RH, et al. (2003) G protein subunit G gamma 13 is coexpressed with G alpha o, G beta 3, and G beta 4 in retinal ON bipolar cells. J Comp Neurol 455: 1-10.

9. Koike C, Obara T, Uriu Y, Numata T, Sanuki R, et al. (2010) TRPM1 is a component of the retinal ON bipolar cell transduction channel in the mGluR6 cascade. Proc Natl Acad Sci U S A 107: 332-337.

10. Witkovsky P, Gábriel R, Krizaj D (2008) Anatomical and neurochemical characterization of dopaminergic interplexiform processes in mouse and rat retinas. J Comp Neurol 510: 158-174.

11. Guo C, Stella SL, Jr., Hirano AA, Brecha NC (2009) Plasmalemmal and vesicular gamma-aminobutyric acid transporter expression in the developing mouse retina. J Comp Neurol 512: 6-26.

12. Coombs J, van der List D, Wang GY, Chalupa LM (2006) Morphological properties of mouse retinal ganglion cells. Neuroscience 140: 123-136.

13. Lim EJ, Kim IB, Oh SJ, Chun MH (2007) Identification and characterization of SMI32-immunoreactive amacrine cells in the mouse retina. Neurosci Lett 424: 199-202.

14. Johnson J, Tian N, Caywood MS, Reimer RJ, Edwards RH, et al. (2003) Vesicular neurotransmitter transporter expression in developing postnatal rodent retina: GABA and glycine precede glutamate. J Neurosci 23: 518-529.

15. Zahir T, Klassen H, Young MJ (2005) Effects of ciliary neurotrophic factor on differentiation of late retinal progenitor cells. Stem Cells 23: 424-432.

16. Barcelona PF, Ortiz SG, Chiabrando GA, Sanchez MC (2011) alpha2-Macroglobulin induces glial fibrillary acidic protein expression mediated by low-density lipoprotein receptor-related protein 1 in Muller cells. Invest Ophthalmol Vis Sci 52: 778-786.

17. Xue L, Ding P, Xiao L, Hu M, Hu Z (2011) Nestin is induced by hypoxia and is attenuated by hyperoxia in Muller glial cells in the adult rat retina. Int J Exp Pathol 92: 377-381.
